# Abdominal apoplexy due to rupture of inferior pancreaticoduodenal artery: A rare case of acute abdomen

**DOI:** 10.22088/cjim.12.0.479

**Published:** 2021

**Authors:** Adel Zeinalpour, Amirhossein Aghili, Barmak Gholizadeh

**Affiliations:** 1 Department of General Surgery , Shahid Beheshti University of Medical Sciences , Tehran , Iran

**Keywords:** Acute Abdomen, Hemoperitoneum, Mesenteric arteries

## Abstract

**Background:**

Abdominal apoplexy is one of the rare causes of non-traumatic intra-abdominal bleeding. This condition is usually seen in male patients in their 50s with history of hypertension. As soon as abdominal apoplexy is suspected, immediate resuscitation should be performed followed by emergent surgery. The patient's outcome depends entirely on the clinical condition and the time interval between diagnosis and treatment.

**Case Presentation:**

Herein we present a 63-year-old man with idiopathic spontaneous intraperitoneal hemorrhage (ISIH) caused by spontaneous rupture of non-aneurysmal inferior pancreaticoduodenalartery (IPDA).

**Conclusion:**

In this report, a case of abdominal apoplexy has been presented caused by spontaneous rupture of non-aneurysmal inferior pancreaticoduodenalartery (IPDA) in a patient without any significant past medical history.

Abdominal apoplexy or “idiopathic spontaneous intraperitoneal hemorrhage (ISIH) is a rare and often fatal clinical condition in which one or more of the major arteries in the abdomen rupture spontaneously, causing intra-abdominal hemorrhage (1, 2). Its causes are not well understood, but it can be in the context of a vascular disorder with damage and weakening of arterial wall which makes the vessel prone to rupture (3). Some patients report a history of high blood pressure, but others do not report any history of specific disease. The clinical manifestations of ISIH are highly variable, but the most common clinical sign is abdominal pain, which is usually generalized due to intra-abdominal hematoma. Other clinical manifestations may include nausea and vomiting, hematochezia, and sometimes shock and hemodynamic instability. On examination, there is usually generalized tenderness with mild to moderate distension in a distressed patient (4, 5). Due to the fact that abdominal apoplexy is a rare disease and difficult to diagnose and requires emergency treatment to save the patient's life. We presented this case caused by spontaneous rupture of non-aneurysmal inferior pancreaticoduodenal artery (IPDA) in a patient without any significant past medical history. 

## Case presentation

A 63-year-old man was referred to Modarres Hospital, Tehran with gradual onset of obscure generalized abdominal pain six hours before admission. His pain was located in the epigastric and periumbilical region without subsequent radiation to anywhere else with one episode of vomiting. 

Other findings were moderate abdominal distension with periumbilical abdominal tenderness without guarding. Initial blood tests showed white cell count (WBC) of 21.7 × 10^9^/L, neutrophils 84.2%, hemoglobin (Hb) 15.2 g/dL, C reactive protein +2 and amylase 63 U/L. abdominal x-ray and ultrasonography were normal.

**Figure 1:**
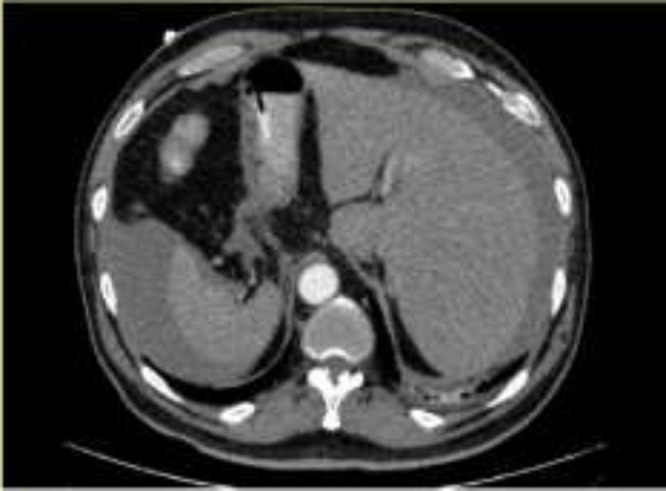
Peri hepatic and Perisplenic intrapritoneal Hemorrhage

**Figure 2:**
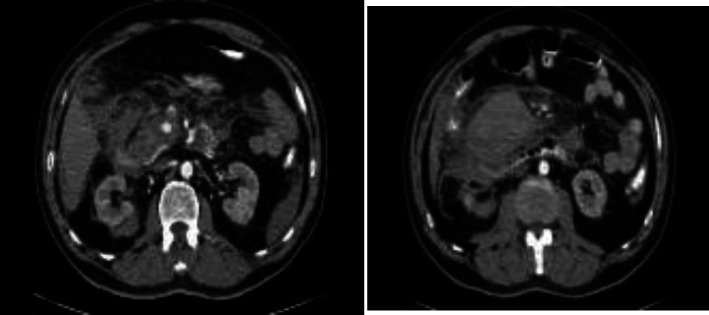
Axial view of Abdominal CT Scan shows Intraperitoneal hematoma

About five hours after resuscitation and appropriate fluid therapy, he has not felt better and blood tests were deranged with WBC of 18.4×10^9^/L, neutrophils 76.5%, Hb 8.1 g/dL and abdominopelvic CT-scan showed evidence of hematoma near the head of pancreas and perihepatic and perisplenic fluid (figures 1, 2). Because of clinical deterioration with a fall in BP to 95/55 mmHg and rise of pulse to 105/min despite appropriate resuscitation, he was transferred to operating room emergently. 

After midline laparotomy and evacuating massive intraperitoneal hematoma about 2.5 liters, right medial visceral rotation, the Cattell-Braasch maneuver, was performed and an active bleeding in the base of IPDA was detected. IPDA was suture ligated at the base with 4/0 nonabsorbable stitch to achieve hemostasis. The post-operative period was uneventful. Oral fluids were started the day after the surgery and patient was discharged from the hospital two days after the surgery. One week after discharge, he had a normal follow-up without any concerning problem.

## Discussion

Abdominal apoplexy is a clinical condition that can occur at any age or sex, but is most common in men between the ages of 50 and 60. Numerous causes can lead to spontaneous intra-abdominal bleeding without a history of trauma, including aneurysms, vasculitis, atherosclerosis, malignancies, and inflammatory diseases. Some patients have history of hypertension (6). Rupture of the inferior pancreaticoduodenal artery is very rare especially in the absence of underlying disease. 

Clinical manifestations of this condition present as the onset of sudden and often severe abdominal pain with or without nausea or vomiting, and sometimes hematochezia. On examination, these patients have abdominal tenderness that does not correlate with clinical condition (6). Bleeding can be intraperitoneal, retroperitoneal or a combination of both (7). First step in the management of patients is based on the clinical and vital stability. In patients presenting with hemodynamic instability and shock, the first step in the management is resuscitation and stabilizing the patient's condition but in unresponsive patients to resuscitative management, surgery may be needed before a definitive diagnosis. If the patient's general condition is stable without deterioration, some diagnostic measures such as abdominal CT scan, could be considered (8). 

The main treatment for ISIH is based on adequate resuscitation and control of bleeding. Usually, emergency laparotomy is performed in these patients but other treatments such as trans-arterial embolization in stable patients have recently been reported (1, 9).

In this patient, because of stable condition, more diagnostic workups were performed. But because of deterioration of his condition, emergency laparotomy was decided for definitive diagnosis and treatment.

Based on our research, this case is a second case of abdominal apoplexy due to spontaneous rupture of non-aneurysmal IPDA in a patient without any significant past medical history. The previous case of abdominal apoplexy due to IPDA was reported by Wang et al. in 2017 (1). The patient was a 55-year-old male with multiple underlying diseases including hypertension. Because of clinical stability, after abdominal CT scan, diagnostic angiography was performed which conﬁrmed diagnosis of abdominal apoplexy and revealed 2 bleeding sites in gastroduodenal artery (GDA) and IPDA. 

Transcatheter embolization was performed (1). Spontaneous inferior pancreaticoduodenal artery rupture is very rare and because of its rarity, it is usually less suspected in abdominal pain, which sometimes causes us to miss it. Therefore, in any patient with abdominal pain, even without a history of other underlying diseases (such as the reported case), we must consider abdominal apoplexy in differential diagnoses. Unfortunately, despite all the advances in imaging, definitive diagnosis of abdominal apoplexy is a major challenge and is usually based on laparotomy. 

In this case report, we emphasize that, in any patient with abdominal pain, even without a history of underlying disease, surgeon must consider abdominal apoplexy in differential diagnoses especially in clinical deterioration of the patient, laparotomy should be considered because in this disease, without immediate surgery, the patient's mortality rate would be very high.
